# Incidence of Malignant Oral Cavity Tumors in the Republic of Serbia: Trend Analysis (1999–2020) and Projections to 2040

**DOI:** 10.3390/medicina62091757

**Published:** 2026-09-11

**Authors:** Vojkan Lazić, Vladimir Videnović, Andrija Ćosić, Milan Miladinović, Marko Matvijenko, Aleksandar Šubarić, Aleksandra Ilić, Vladimir Matvijenko, Dejan Perić, Goran Videnović

**Affiliations:** 1 Department of Dentistry, Faculty of Medicine, University of Pristina in Kosovska Mitrovica, Anri Dinana bb, 38220 Kosovska Mitrovica, Serbia; 2 Department of Radiology, Clinical Hospital Center Zvezdara, Dimitrija Tucovića 161, 11000 Belgrade, Serbia; 3 Department of Maxillofacial Surgery, Faculty of Medicine, University of Niš, Bulevar dr Zorana Đinđića 81, 18000 Niš, Serbia; 4 Department of Preventive Medicine, Faculty of Medicine, University of Pristina in Kosovska Mitrovica, Anri Dinana bb, 38220 Kosovska Mitrovica, Serbia

**Keywords:** oral cavity cancer, incidence trends, joinpoint regression, ARIMA forecasting, cancer registry, Serbia

## Abstract

*Background and Objectives*: Malignant tumors of the oral cavity are an important and growing component of the global cancer burden, with well-documented sex disparities in incidence. This study aimed to characterize temporal trends in the incidence of these tumors in the Republic of Serbia between 1999 and 2020, overall and by sex, and to forecast incidence through 2040. *Materials and Methods*: All histopathologically verified cases (ICD-10: C01–C06, excluding the lip) reported to the Cancer Registry of the Republic of Serbia (excluding the Autonomous Province of Kosovo and Metohija, for which data are unavailable) were analyzed retrospectively. Age-standardized incidence rates (ASRs; world standard population) were calculated, and joinpoint regression estimated the annual percentage change (APC) with 95% confidence intervals (CI). Autoregressive Integrated Moving Average (ARIMA) models projected incidence to 2040, with sensitivity analyses for the 2016 extension of Registry coverage. *Results*: A total of 5695 cases were registered (3872 men, 1823 women). Restricted to the population under consistent Registry coverage (Central Serbia, 1999–2015), incidence did not change significantly in either sex (men: APC = −0.30%, 95% CI: −1.63, 1.06; women: APC = −3.31%, 95% CI: −7.41, 0.96). Over the full 1999–2020 series, which includes the 2016 extension of coverage to Vojvodina, incidence appeared to rise among men (APC = 1.71%; 95% CI: 0.47, 2.96; *p* = 0.009), with an apparent acceleration after 2013 (APC = 8.39%; *p* = 0.002); a level-shift model and a formal comparison of competing trend descriptions both attributed this apparent rise to the 2016 coverage change rather than to a genuine trend (+50.7%; *p* < 0.001). Male incidence was about twice that in women (mean ASR 3.63 versus 1.64). Under an unchanged-coverage scenario, ARIMA models projected a flat mean incidence through 2040 (2.6 per 100,000 overall; 4.7 in men, with a 95% prediction interval widening from 3.6–5.8 in 2021 to 1.2–8.2 in 2040), with a considerably less precise and less reliable forecast for women. *Conclusions*: Oral cavity cancer incidence in Central Serbia was stable from 1999 to 2015, with a persistent male excess. The apparent post-2013 rise is attributable to the 2016 extension of registry coverage rather than to a genuine trend, and a counterfactual estimate suggests considerably higher incidence in Vojvodina. Region-stratified reporting is needed before national trends can be interpreted reliably.

## 1. Introduction

Cancer remains one of the leading causes of morbidity and mortality worldwide: the most recent GLOBOCAN estimates report approximately 20 million new cancer cases and 9.7 million cancer deaths globally in 2022, equivalent to one in five people developing cancer during their lifetime [1]. Within this broader burden, malignant tumors of the lip and oral cavity represent a major and growing global health burden. GLOBOCAN estimated approximately 758,000 new cases of lip, oral cavity and pharyngeal cancer worldwide in 2022, of which the oral cavity alone accounted for about 42% [1,2]. The burden falls unevenly: in countries of lower human development, lip and oral cavity cancer ranks third among men for age-standardized incidence, behind only prostate and lung cancer, at 10.0 per 100,000 [1].

Incidence patterns vary substantially by region, sex, and level of socioeconomic development. Global Burden of Disease analyses have consistently reported higher incidence among men than women across all age groups [3]; however, this male predominance is not static, and recent projections suggest that the global sex gap may narrow over the coming decade, with incidence expected to plateau or decline in men while continuing to rise in women [4]. Whether individual countries follow this global pattern, or diverge from it, remains an open and clinically relevant question.

Tobacco use, in all its forms, is the principal established risk factor for oral cavity cancer. The largest meta-analysis to date, by Possenti and colleagues, pooling 115 original studies and more than 40,000 cases, estimated that current smokers carry an approximately 3.4-fold higher risk of oral cavity cancer than never smokers, with risk rising steeply with both smoking intensity and duration before plateauing at around 20 cigarettes per day or 20 years of smoking [5]; earlier pooled estimates were of comparable magnitude [6]. This excess risk is at least partly reversible: it falls by roughly half within a decade of cessation and approaches that of never smokers after approximately two decades [5,7].

Alcohol consumption is an independent risk factor, and the risk rises with the amount consumed; in a comprehensive meta-analysis, heavy drinkers had an approximately five-fold higher risk of oral and pharyngeal cancer than non-drinkers or occasional drinkers, and an excess risk was detectable even at low intakes [8]. Alcohol exposure is itself strongly sex-patterned: within the WHO European Region, which records the highest per-capita consumption of any WHO region, men consume almost four times as much pure alcohol per year as women (14.9 versus 4.0 L) [9]. When tobacco and alcohol exposures coincide, their joint effect exceeds the product of the individual effects [10,11]. In the pooled analysis of the International Head and Neck Cancer Epidemiology (INHANCE) consortium, 64% of oral cavity cancers were attributable to tobacco or alcohol use, and this attributable fraction was substantially higher in men (74%) than in women (57%) [11]. That difference is directly relevant to the sex-specific patterns examined here.

Other factors have been implicated, most prominently human papillomavirus (HPV) infection, poor oral health, and low intake of fruit and vegetables [12]. The evidence for HPV is, however, considerably stronger for the oropharynx than for the oral cavity proper. Pooled HPV DNA prevalence in oral cavity carcinoma is approximately 26%, with wide between-study heterogeneity [13]. When expression of the HPV E6/E7 oncogenes is required instead of mere DNA presence, however, the etiologic fraction attributable to high-risk HPV in oral cavity carcinoma falls to about 6% [14]. Periodontitis has likewise been associated with increased oral cancer risk in a meta-analysis of case-control studies (OR = 2.94; 95% CI: 2.13, 4.07), although the certainty of the evidence was rated low and heterogeneity between studies was substantial [15].

Central and Eastern Europe is among the higher-burden regions globally for lip, oral cavity, and pharyngeal cancers [16], a pattern consistent with the historically high prevalence of tobacco and alcohol use across the region. This regional excess is long-standing: whereas oral and pharyngeal cancer mortality began to decline in southern and western Europe during the 1990s, several Central and Eastern European countries recorded persistent increases over the same period, with rates in Hungary and Slovakia among the highest ever documented [17]. The scale is easily underestimated. Among Hungarian men aged 35 to 64, mortality from these cancers reached 55.3 per 100,000, comparable to lung cancer mortality in several major European countries [17]. Subsequent analyses of global incidence and mortality trends confirm that this east–west gradient has persisted into the last decade [18,19], and projections based on Global Burden of Disease data identify Eastern Europe as one of the regions in which the burden of lip and oral cavity cancer is expected to continue rising towards 2040 [20].

Within Serbia, several registry-based studies have examined related head and neck cancer sites, including oral cavity and pharyngeal cancer combined at the national level [21] and specifically within the city of Belgrade for the period 1999–2010 [22], “other and ill-defined” sites of the lip, oral cavity, and pharynx [23], and laryngeal cancer [24], typically using joinpoint regression to characterize temporal trends. National survey data indicate that tobacco use remains highly prevalent in Serbia, with 31.9% of those aged 15 years and over using tobacco products in 2019, and that the decline in daily smoking since 2000 has been far steeper in men than in women [25]; tobacco and alcohol use also frequently co-occur in the same individuals: among Serbian adults who binge drink frequently, smoking prevalence exceeds two thirds [26]. However, no prior study has specifically isolated the oral cavity subsite (ICD-10: C01–C06, excluding the lip) to characterize its sex-specific incidence trend in Serbia over a long observation period, or combined trend analysis with quantitative forecasting to project future disease burden.

This study therefore aimed to (1) describe temporal trends in the incidence of malignant oral cavity tumors in the Republic of Serbia between 1999 and 2020, overall and by sex, using joinpoint regression analysis; (2) forecast oral cavity cancer incidence through 2040 using ARIMA time-series modeling, in order to inform future cancer control planning; and (3) test the robustness of the observed trends to changes in Registry coverage.

## 2. Materials and Methods

### 2.1. Study Design, Data Source, and Study Population

This retrospective, population-based study used data from the Cancer Registry of the Republic of Serbia (the Registry). Cancer reporting is mandatory by law in Serbia [27], and the Registry methodology follows the recommendations of the International Association of Cancer Registries (IACRs) and the European Network of Cancer Registries (ENCRs), of which the Registry became a full member in 1998; since 2014, the national Registry has been maintained by the Institute of Public Health of Serbia [28,29].

We identified all histopathologically verified cases of malignant oral cavity tumors (ICD-10: C01–C06, excluding lip tumors) reported to the Registry between 1 January 1999 and 31 December 2020. A total of 5695 patients were registered (3872 men and 1823 women). The geographical coverage of the Registry changed during the study period, and this is central to the interpretation of our results. For diagnoses up to and including 2015, the reported cases cover Central Serbia only, excluding the Autonomous Province of Vojvodina. From 2016 onwards, they cover Central Serbia together with Vojvodina. The Autonomous Province of Kosovo and Metohija is excluded throughout the whole study period. This change is documented in the Registry’s own publication series, which reported on Central Serbia for the years up to 2015 [28] and on the Republic of Serbia as a whole from 2016 onwards [29]. Annual age-standardized incidence rates were calculated against the corresponding resident population for each period: the population of Central Serbia for 1999–2015, and of Central Serbia plus Vojvodina for 2016–2020. Each annual rate is therefore internally consistent with its own numerator. Annual population estimates and migration data were obtained from the Statistical Office of the Republic of Serbia; according to those estimates, Vojvodina accounts for approximately 26% of the population of the combined area [30]. Case counts disaggregated by region, which would have permitted the two areas to be analyzed separately, were not available to the authors.

### 2.2. Case Definition and Classification

Cases were classified according to the International Classification of Diseases, 10th Revision (ICD-10) [31].

Malignant tumors of the oral cavity (ICD-10: C01–C06) comprised the following anatomical subsites: tongue (C01–C02), gum (C03), floor of mouth (C04), palate (C05), and other and unspecified parts of the mouth, including the buccal mucosa (C06). Malignant tumors of the lip (C00) and pharynx (C09–C10, C12–C14) were analyzed separately and are not part of the present study. Malignant tumors of the nasopharynx (C11), nasal cavity (C30.0), and skin of the lip (C44.0) were excluded owing to their distinct etiopathogenesis. For brevity, the terms “oral cavity cancer” and “malignant oral cavity tumors” are used interchangeably throughout this manuscript to denote the same case definition (ICD-10: C01–C06, excluding lip).

### 2.3. Statistical Analysis

#### 2.3.1. Incidence Rates

Age-standardized incidence rates (ASRs) were calculated by direct standardization, using the world standard population as the reference [32]. Rates are expressed per 100,000 person-years. All analyses were performed separately for men and women in addition to the combined series, and sex-disaggregated data are reported throughout; sex refers to the biological attribute as recorded in the Registry.

#### 2.3.2. Trend Analysis (Joinpoint Regression)

Temporal trends in incidence and the annual percentage change (APC), with corresponding 95% confidence intervals (CIs), were estimated using joinpoint regression analysis [33], performed in the Joinpoint Regression Program, version 5.0.2 (Statistical Methodology and Applications Branch, Surveillance Research Program, National Cancer Institute, Bethesda, MD, USA). A maximum of four joinpoints was permitted, and the location of each joinpoint was reported with its 95% confidence interval. A *p*-value < 0.05 was considered statistically significant.

#### 2.3.3. Forecasting (ARIMA Modeling)

To project oral cavity cancer incidence for the subsequent 20 years (2021–2040), separate Autoregressive Integrated Moving Average (ARIMA) models were fitted to incidence data from 1999–2020 for both sexes combined and for men and women individually.

Model development followed a stepwise procedure: (a) stationarity was assessed using the Augmented Dickey–Fuller (ADF) test, and the series was differenced as needed (parameter *d*) until stationarity was achieved; (b) the autoregressive (*p*) and moving-average (*q*) orders were identified by inspection of the autocorrelation function (ACF) and partial autocorrelation function (PACF); to minimize subjective bias, final model order selection was performed using the automated auto.arima function in R, with the optimal ARIMA (*p*,*d*,*q*) model chosen based on the lowest corrected Akaike Information Criterion (AICc) and Bayesian Information Criterion (BIC) values, with AICc prioritized to balance goodness of fit against parsimony; model performance was assessed using the root mean square error (RMSE), mean absolute error (MAE), and mean absolute percentage error (MAPE); and (c) model adequacy was validated by testing residuals for normality (Shapiro–Wilk test) and autocorrelation (Ljung–Box test).

All time-series analyses were performed in R, version 4.5.2 (R Foundation for Statistical Computing, Vienna, Austria), using the forecast [34], tseries, lmtest, ggplot2, dplyr, Metrics, and gridExtra packages. A *p*-value < 0.05 was considered statistically significant.

#### 2.3.4. Sensitivity Analyses for the Change in Registry Coverage

The extension of Registry coverage in 2016 alters the geographical composition of the series. Three sensitivity analyses were therefore performed to test whether the estimated trends are attributable to that change. First, log-linear regression of the age-standardized rate on calendar year was refitted to the truncated series 1999–2015, the period of consistent coverage. Second, an indicator variable for 2016 onwards was added to a log-linear model of the full series. This allows a multiplicative level shift at the point of the coverage change to be estimated separately from the underlying annual trend. Both were implemented by the authors as ordinary least-squares regressions on log-transformed rates, in Python 3.12 with NumPy. They were run outside the Joinpoint Regression Program, which does not accommodate a known structural break of this kind. The magnitude of the discontinuity was then used to derive the incidence implied for Vojvodina by solving the population-weighted identity that relates the combined-area rate to the rates of its two constituent areas. The rate for Central Serbia in 2016–2020 was taken to be its mean over 2013–2015, the last three years of consistent coverage, and the population weight for Vojvodina was 26%. Because this calculation rests on the counterfactual assumption that Central Serbia’s rate would have remained at its 2013–2015 level after 2015, a sensitivity analysis was performed in which this assumption was relaxed: the Central Serbia rate for 2016–2020 was instead projected forward from 2015 using the point estimate and 95% confidence bounds of the male and combined-sex annual percentage change from the consistent-coverage joinpoint model (1999–2015), and the implied Vojvodina rate was recalculated under each trajectory. Third, three competing descriptions of each series were compared directly using the Bayesian Information Criterion (BIC): a single log-linear trend, the best-fitting continuous one-joinpoint model with the break position selected by an exhaustive search over all candidate years, and a model combining a single trend with a discontinuous change in level at 2016. Lower BIC indicates a better description. Annual case counts, population denominators, and code reproducing the ARIMA models are provided in Supplementary Table S1 and Code S1.

### 2.4. Use of Generative Artificial Intelligence

Generative artificial intelligence (Claude Sonnet 5, Anthropic) was used for language editing.

### 2.5. Ethical Approval

This study forms part of a broader research project conducted within the doctoral dissertation of the first author, approved by the Ethics Committee of the Faculty of Medicine, University of Priština in Kosovska Mitrovica (Decision No. 09-962-1, dated 5 June 2018). The analysis presented in this manuscript is based on aggregated, fully anonymized data from the Cancer Registry of the Republic of Serbia, without access to individual-level patient data; the research was conducted in accordance with the principles of the Declaration of Helsinki.

## 3. Results

### 3.1. Case Distribution and Incidence Rates, 1999–2020

During the study period, 5695 patients with malignant oral cavity tumors (ICD-10: C01–C06, excluding lip tumors) were registered, comprising 3872 men (68.0%) and 1823 women (32.0%). The annual number of cases ranged from 156 (2011) to 443 (2019), with a mean of 259 cases per year (mean 176 men and 83 women annually). Case counts and age-standardized incidence rates (ASRs, world standard population) by year and sex are presented in Table 1.

The overall (both-sex) ASR ranged from 1.68 to 3.98 per 100,000 (mean 2.57). Among men, ASRs ranged from 2.49 (2011) to 5.00 per 100,000 (2017–2018, mean 3.63); among women, ASRs ranged from 0.89 (2010) to 4.51 per 100,000 (2003, mean 1.64).

Cases were further classified by anatomical subsite. Among the 5695 oral cavity cancers, the tongue was the most frequently affected subsite (2469 cases, 43.4% of the total), followed by other and unspecified parts of the mouth (1211; 21.3%), the floor of the mouth (1156; 20.3%), the palate (549; 9.6%), and the gum (310; 5.4%) (Table 2). The sex distribution differed significantly across subsites (χ^2^ = 74.2, *p* < 0.001): the tongue was disproportionately affected in men (76.1% male, versus 68.0% male overall), whereas other and unspecified parts of the mouth were the only subsites in which women outnumbered men (54.3% female).

### 3.2. Primary Analysis: Trends in the Population with Consistent Registry Coverage, 1999–2015

The number of registered cases rose from a mean of 217 per year in 2013–2015 to 417 per year in 2016–2020, an increase of 92%, while the reference population increased by roughly a third with the addition of Vojvodina. The age-standardized rate rose across the same break by 32% overall and by 43% among men. Had incidence been equal in the two areas, extending coverage would have left the age-standardized rate unchanged, because the numerator and denominator would have increased in the same proportion.

When the series was restricted to 1999–2015, the period of consistent coverage, the trend among men was flat and not statistically significant (APC = −0.30; 95% CI: −1.63, 1.06; *p* = 0.666). The same was true among women (APC = −3.31; 95% CI: −7.41, 0.96; *p* = 0.127) and for both sexes combined (APC = −1.43; 95% CI: −3.43, 0.60; *p* = 0.166). The level-shift model fitted to the full series gave closely concordant estimates of the underlying trend. It also identified a large and statistically significant discontinuity at 2016, both for men (+50.7%; 95% CI: 25.5, 81.1; *p* < 0.001) and for both sexes combined (+51.6%; 95% CI: 15.3, 99.3; *p* = 0.003). Table 3 presents both analyses.

Solving the population-weighted identity for the combined area gives a counterfactual estimate of the incidence implied for Vojvodina during 2016–2020, under the assumption that Central Serbia’s rate remained at its 2013–2015 level: approximately 8.7 per 100,000 among men, against 3.4 in Central Serbia, a 2.6-fold difference; 2.0 against 1.4 among women, a 1.4-fold difference; and 5.1 against 2.3 overall. This assumption was tested in a sensitivity analysis (Table 4): allowing Central Serbia’s rate to instead follow the point estimate or 95% confidence bounds of the observed 1999–2015 trend changes the implied Vojvodina rate and the size of the gap, but not its direction—under every trajectory tested, the counterfactual Vojvodina rate exceeds that of Central Serbia by a factor of 1.9 to 2.9 among men and 2.1 to 2.7 for both sexes combined.

The joinpoint models themselves point in the same direction. The Joinpoint software was permitted up to four joinpoints, and in no stratum, at any model order, did it place a break at 2015 or 2016. Instead, when additional joinpoints were allowed, the fitted trend split into a steep climb followed by a flat or falling segment. In men, for example, the two-joinpoint model gave an APC of −1.12 for 1999–2013, +14.30 for 2013–2017 and −2.73 for 2017–2020, none of which reached statistical significance. That shape is what a single abrupt change in level looks like when it is forced through a model that must remain continuous.

A direct comparison of competing descriptions makes the point formally (Table 5). For both sexes combined and for men, a model consisting of a single trend plus a discontinuous change in level at 2016 fitted better than the best continuous one-joinpoint model, despite using fewer parameters (BIC −3.0622 versus −2.9453, and −3.8591 versus −3.7131, respectively). For women, neither a joinpoint nor a level shift improved on a simple log-linear trend, consistent with a flat and noisy series. On this evidence, the pattern in the male and combined series is better described as a one-time jump in 2016 than as a change in the underlying trend.

### 3.3. Trends Across the Full Study Period, 1999–2020 (Secondary Analysis, Confounded by the 2016 Coverage Change)

For comparison with published data—which typically report national trends without adjusting for changes in registry coverage—this section presents the unsegmented joinpoint analysis of the full 1999–2020 series. As shown in Section 3.2, this analysis is confounded by the 2016 extension of coverage and should not be interpreted as evidence of a genuine change in incidence.

**Figure 1:**
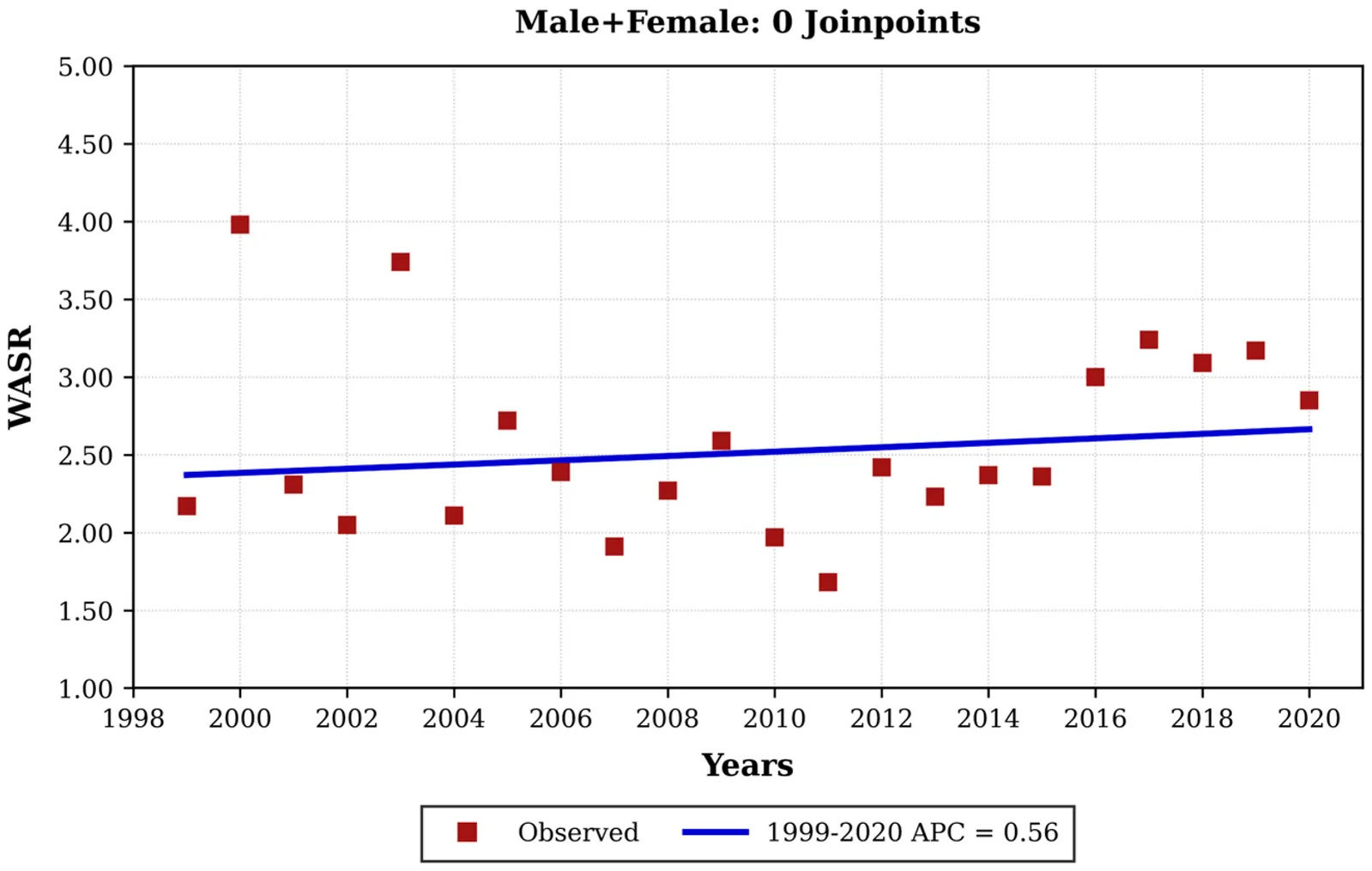
Incidence trend of malignant oral cavity tumors (ICD-10: C01–C06), both sexes, 1999–2020 (0 joinpoints). WASR—world age-standardized rate, the term used by the Joinpoint Regression Program; equivalent to ASR in the text.

**Figure 2:**
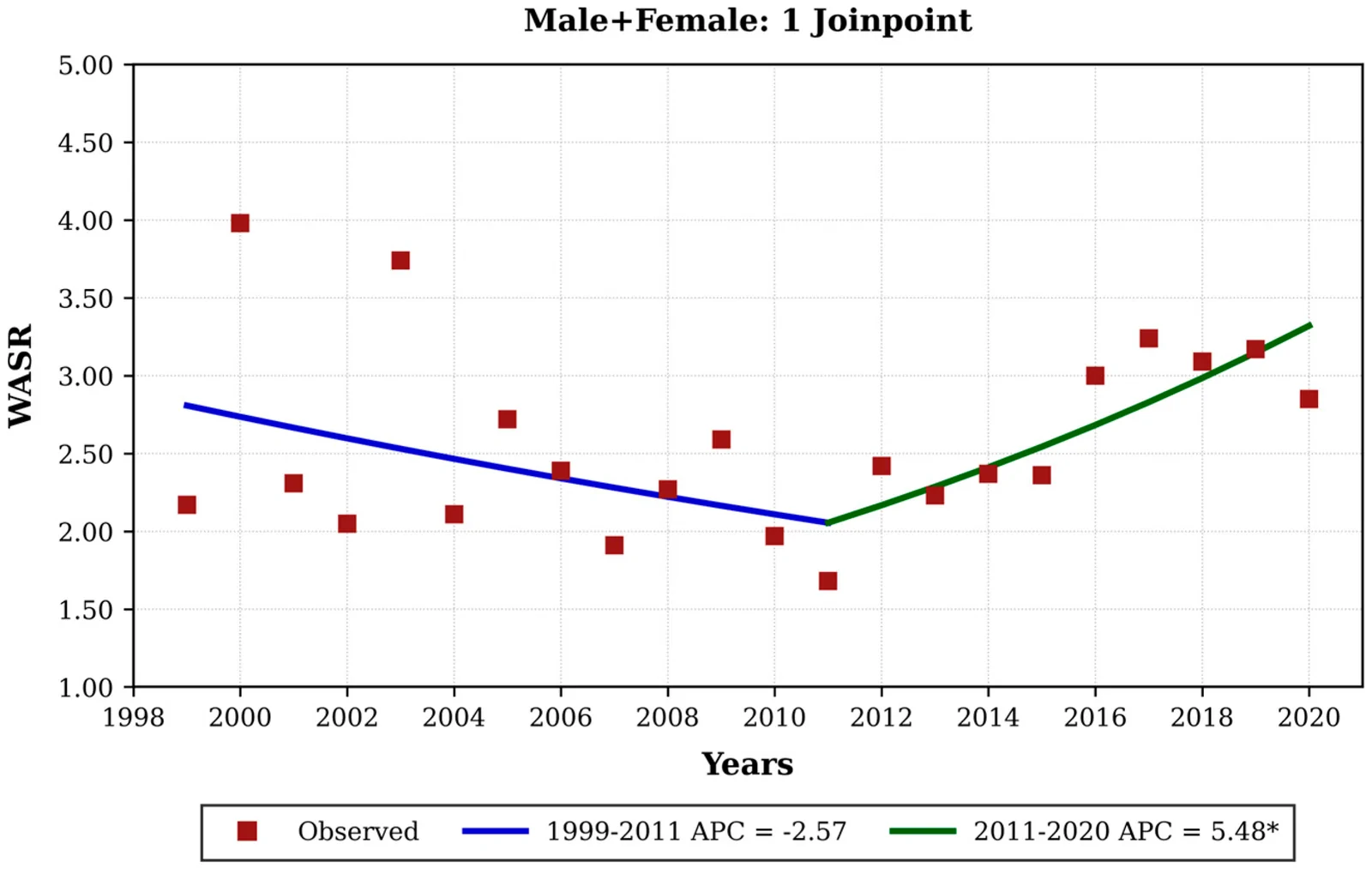
Joinpoint regression model of malignant oral cavity tumor incidence, both sexes, 1999–2020 (1 joinpoint, 2011). WASR—world age-standardized rate, the term used by the Joinpoint Regression Program; equivalent to ASR in the text. * statistically significant (*p* < 0.05).

For both sexes combined, incidence showed a non-significant upward tendency over the entire study period (APC_1999–2020_ = 0.56; 95% CI: −1.00, 2.14) (Figure 1). A single joinpoint was identified in 2011 (95% CI: 2005, 2016), separating a non-significant decline from 1999–2011 (APC = −2.57; 95% CI: −5.77, 0.74) from a statistically significant increase from 2011–2020 (APC = 5.48; 95% CI: 0.18, 11.05; *p* = 0.043) (Figure 2).

Among men, incidence increased significantly across the full study period (APC_1999–2020_ = 1.71; 95% CI: 0.47, 2.96; *p* = 0.009), driven by a marked and statistically significant acceleration after a joinpoint in 2013 (95% CI: 2009, 2015; APC_2013–2020_ = 8.39; 95% CI: 3.44, 13.57; *p* = 0.002), following a non-significant decline from 1999–2013 (APC = −0.64; 95% CI: −2.26, 1.00).

**Figure 3:**
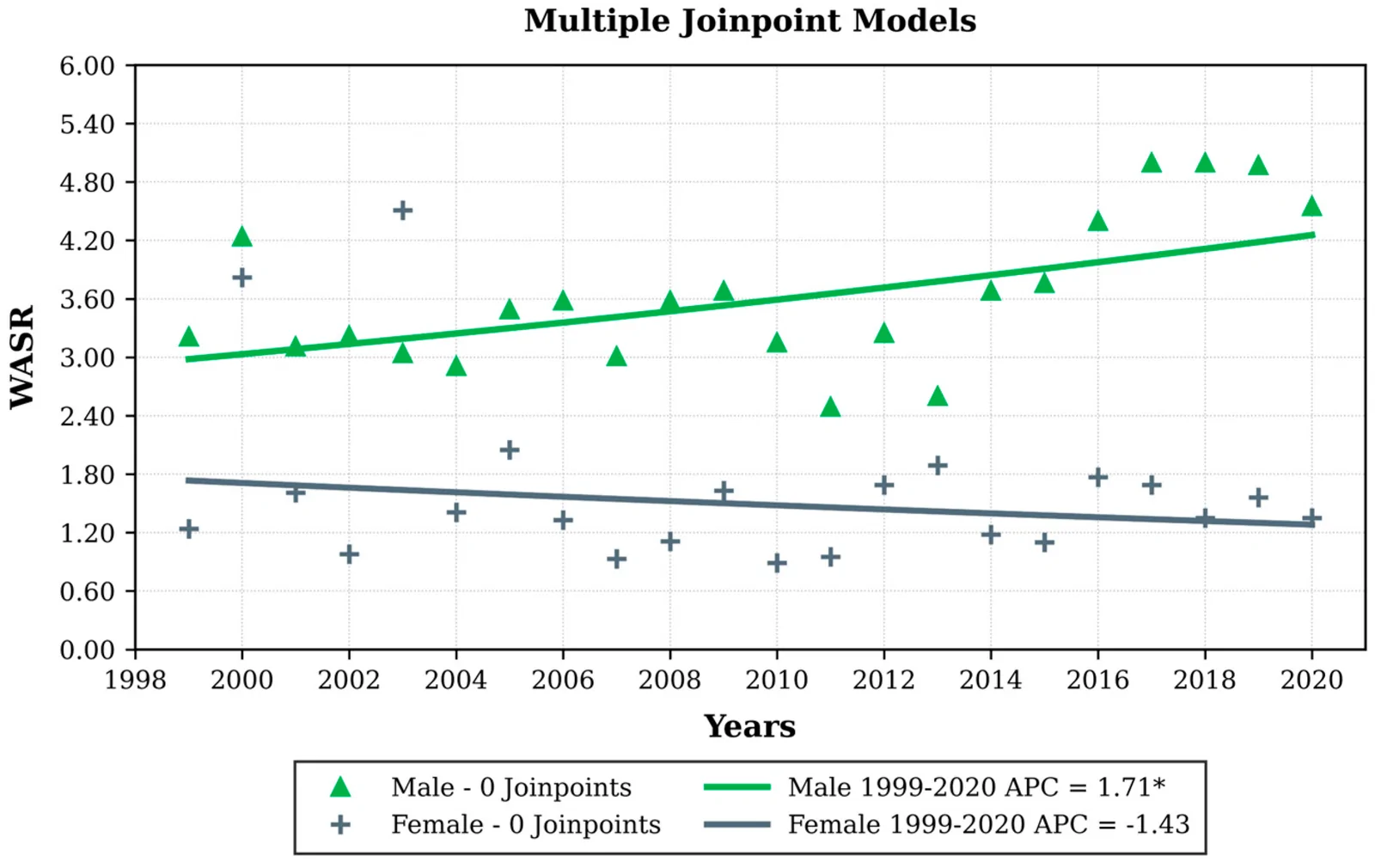
Comparative incidence trends of malignant oral cavity tumors by sex, 1999–2020. WASR—world age-standardized rate, the term used by the Joinpoint Regression Program; equivalent to ASR in the text. * statistically significant (*p* < 0.05).

In contrast, women showed a non-significant decline in incidence across the study period (APC_1999–2020_ = −1.43; 95% CI: −4.21, 1.43; *p* = 0.306), and no statistically significant joinpoint (the joinpoint was estimated at 2010 but with a 95% confidence interval spanning 2001 to 2018; 1999–2010: APC = −5.13, 95% CI: −12.64, 3.02; 2010–2020: APC = 2.99, 95% CI: −6.36, 13.28). This divergence between the sexes is illustrated in Figure 3.

Full results of the joinpoint regression analysis, including the annual percentage change with 95% confidence intervals, are summarized in Table 6. These estimates are re-examined in Section 3.2 in light of the change in Registry coverage.

### 3.4. Forecast of Incidence to 2040 (ARIMA Modeling)

**Figure 4:**
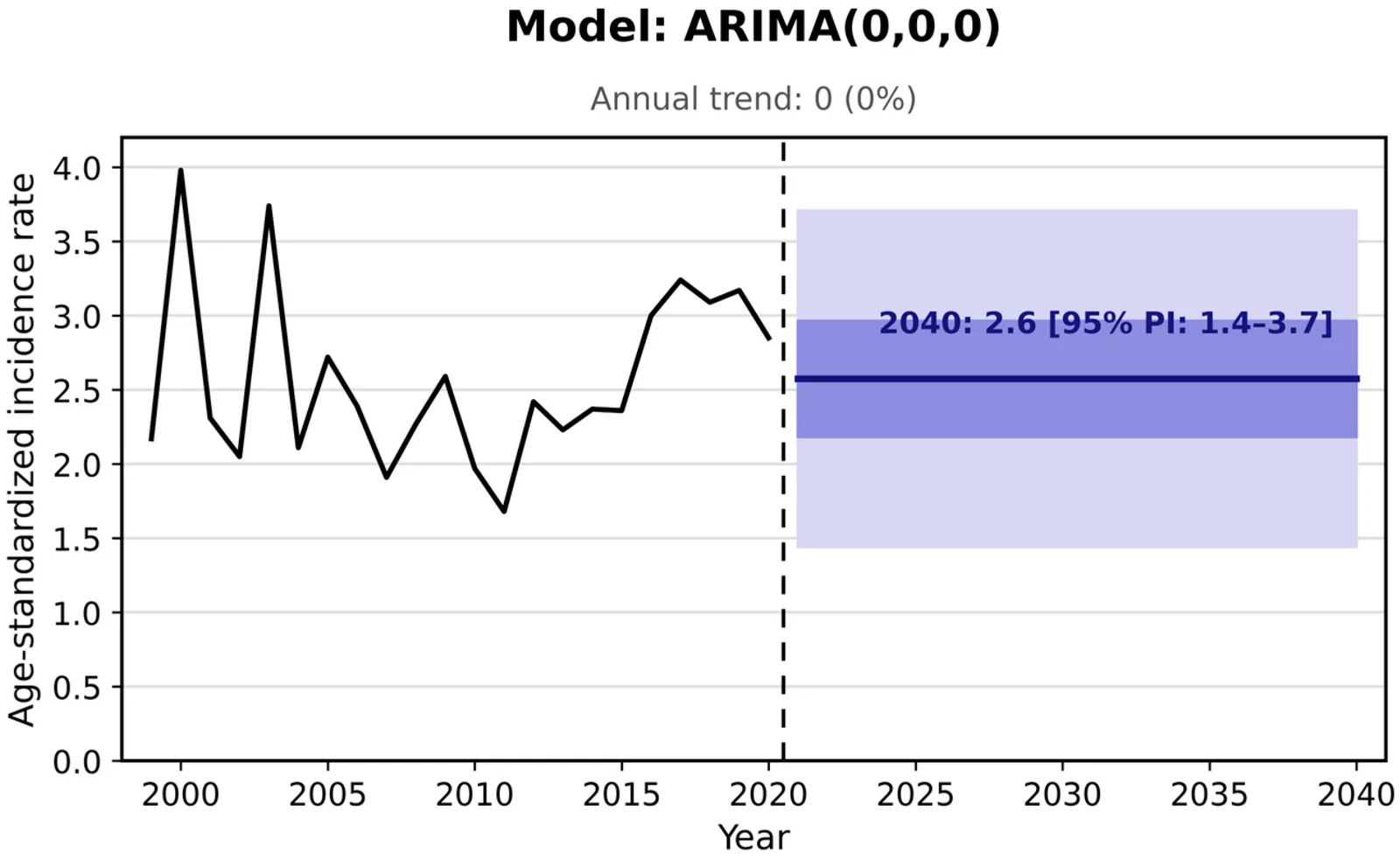
ARIMA(0,0,0) forecast of malignant oral cavity tumor incidence, both sexes, 2021–2040. Dark shading, 50% prediction interval; light shading, 95% prediction interval. Dashed vertical line, boundary between observed (1999–2020) and forecast (2021–2040) periods.

For both sexes combined, the 1999–2020 time series had a mean ASR of 2.6 ± 0.6 (95% CI: 2.3–2.8; range 1.7–4.0). The optimal model, ARIMA(0,0,0) (AICc = 43.0; BIC = 44.5), showed acceptable fit (MAPE = 18.6%; RMSE = 0.578; MAE = 0.474), with normally distributed residuals (Shapiro–Wilk W = 0.934, *p* = 0.145) and no residual autocorrelation (Ljung–Box χ^2^ = 5.710, *p* = 0.336). Under a no-intervention, unchanged-coverage scenario, the model projects a flat mean of 2.6 per 100,000 through 2040 (95% prediction interval [PI]: 1.4–3.7), with no evidence of a directional trend (Figure 4).

**Figure 5:**
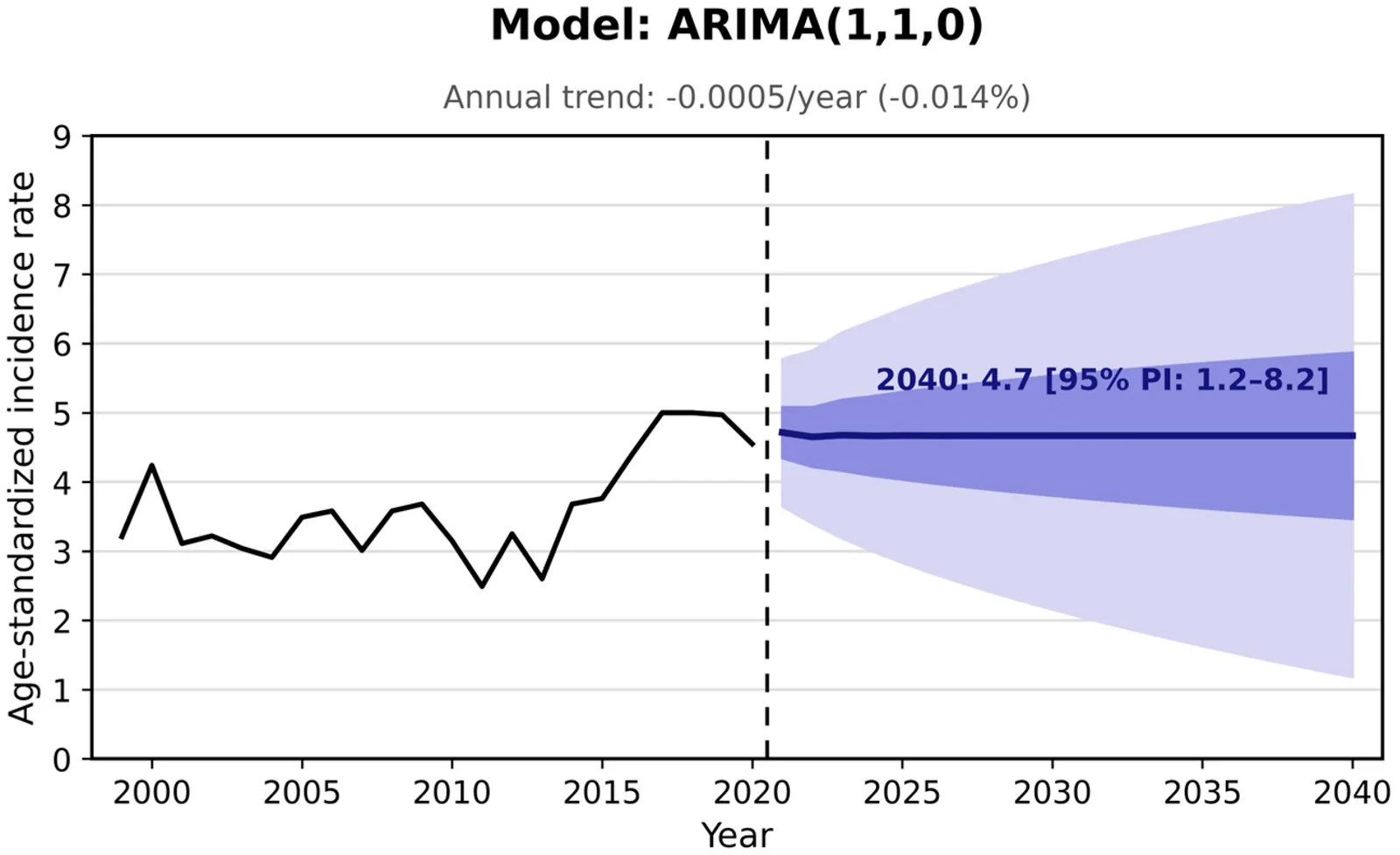
ARIMA(1,1,0) forecast of malignant oral cavity tumor incidence, men, 2021–2040. Dark shading, 50% prediction interval; light shading, 95% prediction interval. Dashed vertical line, boundary between observed (1999–2020) and forecast (2021–2040) periods.

For men, the mean ASR was 3.6 ± 0.8 (95% CI: 3.3–3.9; range 2.5–5.0). The optimal model, ARIMA(1,1,0) (AICc = 38.8; BIC = 40.3), showed good fit (MAPE = 13.2%; RMSE = 0.531; MAE = 0.462), with no residual autocorrelation (Ljung–Box χ^2^ = 2.369, *p* = 0.796) and normally distributed residuals (Shapiro–Wilk W = 0.961, *p* = 0.512). The model projects a mean of 4.7 per 100,000 by 2040, with a negligible negative trend (−0.0005/year; −0.014%); however, the 95% PI widens from 3.6–5.8 in 2021 to 1.2–8.2 in 2040, reflecting the limited information available from only five years of post-2016 data, and the forecast should be read as a scenario under current conditions rather than a precise prediction (Figure 5).

**Figure 6:**
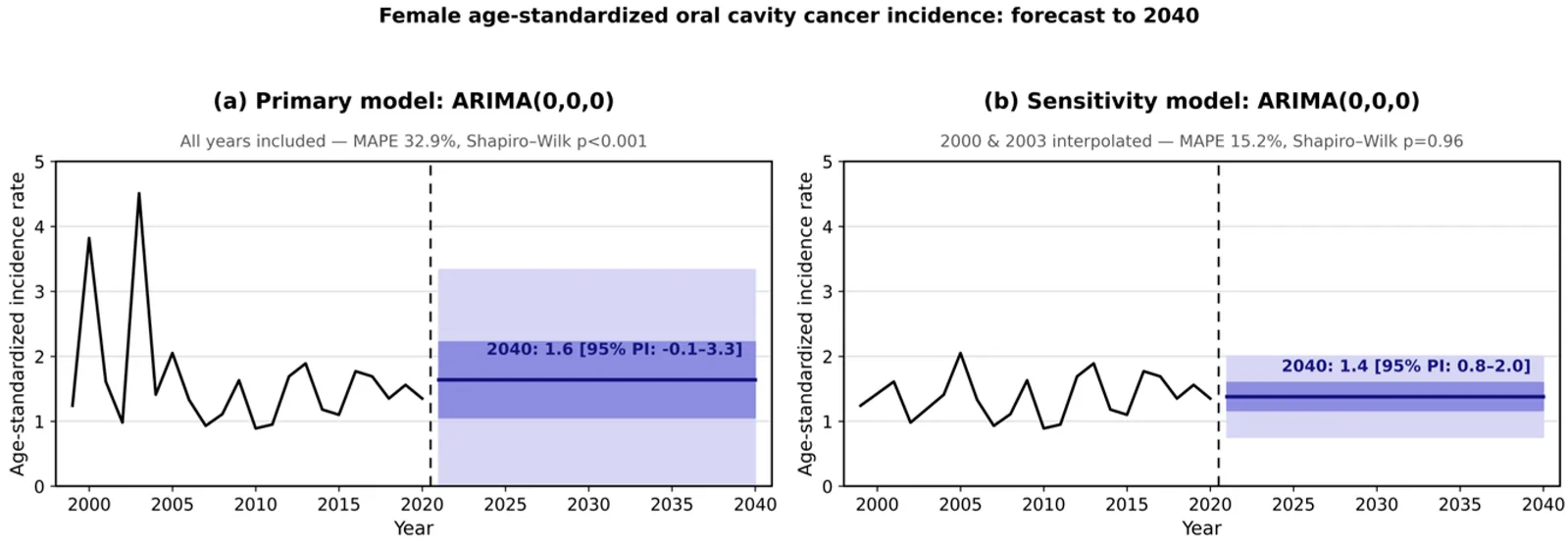
Female age-standardized oral cavity tumor incidence, forecast to 2040. (**a**) Primary ARIMA(0,0,0) model fitted to the full 1999–2020 series, including two anomalous years (2000, 2003) with implausibly high female case counts; the 95% prediction interval for 2040 includes negative values. (**b**) Sensitivity model with the 2000 and 2003 values replaced by linear interpolation from adjacent years; model fit and residual normality improve substantially, and the 2040 prediction interval no longer includes implausible values. Dark shading, 50% prediction interval; light shading, 95% prediction interval. Dashed vertical line, boundary between observed (1999–2020) and forecast (2021–2040) periods.

For women, the mean ASR was 1.6 ± 0.9 (95% CI: 1.2–2.0; range 0.9–4.5), with marked variability (coefficient of variation, CV = 54.1%). The optimal model, ARIMA(0,0,0) (AICc = 60.7; BIC = 62.3), showed weaker fit (MAPE = 32.9%; RMSE = 0.866; MAE = 0.541); residuals departed from normality (Shapiro–Wilk W = 0.691, *p* < 0.001), although no statistically significant residual autocorrelation was detected (Ljung–Box χ^2^ = 5.192, *p* = 0.393). This poor fit is attributable in large part to two anomalous years, 2000 and 2003, in which female case counts (140 and 156) were two to three times those of surrounding years and, unusually, exceeded male counts in 2003—a pattern consistent with a sex-coding error rather than a true incidence spike (see Discussion). The unadjusted model projects an unstable mean incidence of 1.6 per 100,000 through 2040, with a 95% PI (−0.1–3.3) that implausibly includes negative values. In a sensitivity analysis in which the 2000 and 2003 values were replaced with locally interpolated estimates, model fit improved substantially (MAPE = 15–21%; Shapiro–Wilk *p* = 0.64–0.96; coefficient of variation falling from 54.1% to 23.7%), and the 2040 PI narrowed to a plausible range (0.8–2.0) that excludes negative values. Given this sensitivity, the female forecast (Figure 6) should be read as indicative only, pending verification of the 2000 and 2003 records with the Registry.

Model validation parameters for all three ARIMA models are summarized in Table 7.

## 4. Discussion

Between 1999 and 2020, 5695 people in the area covered by the Registry were diagnosed with cancer of the oral cavity. Two of every three were men. Across the whole period, recorded incidence rose in men and stayed flat in women. The rise in men, however, cannot be taken at face value, because the area covered by the Registry changed in 2016. Once that change is accounted for, the underlying trend is flat in both sexes. One finding does not depend on it: men were diagnosed about twice as often as women. The mean age-standardized rate was 3.63 per 100,000 in men against 1.64 in women, and male rates exceeded female rates in 21 of the 22 years.

A large sex gap is not unique to Serbia. Global Burden of Disease analyses report higher rates in men than in women in every age group [3]. They also show incidence rising overall while mortality falls, with the burden spread unevenly across levels of socioeconomic development [35]. Central and Eastern Europe is among the most heavily burdened regions in the world for cancers of the lip, oral cavity and pharynx, behind only Melanesia and South-Central Asia [16], and the difference between eastern and western Europe has been recognized for decades [17,18].

GLOBOCAN estimates for 2022 put the global age-standardized incidence of oral cavity cancer at 4.6 per 100,000 in men and 2.0 in women [2]. Our figures for 2016–2020 were 4.8 and 1.5. Serbian men therefore sit at the world average and Serbian women below it. Both are far below the highest-incidence countries: in men, 13.0 in India, 12.4 in Sri Lanka and 12.0 in Bangladesh, where smokeless tobacco and areca nut are the main causes [2]. One qualification applies: our case definition includes the base of tongue (C01), which the GLOBOCAN subsite analysis assigns to the oropharynx [2], so our rates are marginally higher than a like-for-like figure would be.

The European picture depends on which subsites are counted. Diz and colleagues reported rates separately by ICD-10 subsite, which allows C01–C06 to be isolated [36]. On that measure, Serbia ranks low: male rates were 9.8 in Croatia, 9.6 in Slovakia, 8.7 in Slovenia and in Spain and 5.9 in Italy, against 3.8 in Serbia, with only Poland and Cyprus lower [36]. Female rates place Serbia lower still, at 1.1, against 2.4 in Slovenia, Spain, Italy and Malta [36]. The Serbian sex ratio is therefore wide not because male rates are high but because female rates are low. On Diz’s figures, Croatia reaches almost eight to one on high male rates and Serbia about three to one, on low rates in both sexes. This looks contradictory, since Serbia ranks among the more heavily burdened European countries when lip, oral cavity and pharyngeal cancers are counted together, at 11.7 per 100,000 [36]. The explanation is straightforward: the Serbian burden at these sites lies largely in the lip and the pharynx rather than the oral cavity, and this study covers neither.

The comparison with the larynx is interesting for one reason. Nešić and colleagues reported a mean age-standardized incidence of laryngeal cancer in Central Serbia of 11.1 per 100,000 in men and 1.4 in women [24], a ratio close to eight to one against roughly two to one in our data. Both sites share tobacco and alcohol as their principal causes, so a sex ratio that differs this much suggests that other factors carry more weight in the oral cavity, particularly among women.

One earlier Serbian study is directly comparable to ours. Videnović and colleagues reported C01–C06 separately, with the same exclusions, within a broader analysis of lip, oral cavity and pharyngeal cancers in the Belgrade population for 1999–2010, and recorded male age-standardized incidence rising from 3.75 to 7.23 per 100,000 [22]. Belgrade lies within Central Serbia, and the two findings are best read as complementary: the Belgrade series covers a shorter period and a largely urban population, whereas ours covers the whole area over 22 years. Trends in a capital city need not follow those of the surrounding region, and internal differences of exactly this kind are what the present study identifies as deserving separate analysis. Other Serbian analyses covered oral cavity and pharyngeal cancer combined at the national level [21], or the less well-defined sites within the group [23], and are not directly comparable.

Why was incidence in our data about twice as high in men as in women? Our data cannot answer that directly, since the Registry records no individual-level exposure, but the literature and national surveys provide a pattern our finding fits. Tobacco and alcohol remain the two main causes of oral cavity cancer, and their joint effect is multiplicative or more than multiplicative—that is, greater than the sum of the two acting separately [10,11]. What matters here is that they do not explain equally much in both sexes: in the pooled INHANCE analysis, Hashibe and colleagues attributed 74% of head and neck cancers in men to tobacco and alcohol but only 57% in women, and 64% for the oral cavity specifically [11]. In men, then, most of these cancers can be traced to smoking and drinking; in women, almost half cannot.

National surveys show exactly that difference in habits. Daily smoking among men fell from 46.0% in 2000 to 29.4% in 2019, while among women it barely moved, from 26.1% to 25.0% [25]. Drinking follows the same pattern: in 2019, 5.7% of men drank daily against 0.7% of women, and 3.2% of men reported heavy drinking at least weekly against 0.3% of women [25]. The two habits travel together—among adults who binge drink often, more than two-thirds smoke [26]—and Serbia lies in the WHO region with the highest per-head alcohol consumption in the world [9], where high rates are attributed to spirits and home-produced beverages rich in acetaldehyde [18].

Timing adds to this. Oral cavity cancer appears 15 to 20 years after the exposure that caused it, and risk falls slowly after quitting: by about half after ten years, and close to that of a never-smoker only after twenty [5,7]. The benefit of the post-2000 fall in male smoking would therefore not yet be visible by 2020, and cases diagnosed in the 2010s largely reflect smoking done in the 1980s and 1990s. In women, the picture differs: smoking prevalence barely changed, so neither benefit nor harm would be expected over this period, and the female trend was mildly downward in every analysis but statistically significant in none (APC = −1.43 across the whole period, −3.31 over the years of consistent coverage). None of this establishes cause, but it fits an international pattern: across 37 countries, Simard and colleagues found oral cavity incidence rising where tobacco epidemics were still peaking, falling where they had peaked earlier, and stable in most of the rest [37].

The apparent acceleration among men after 2013 (APC = 8.39; *p* = 0.002) must be read in the light of the coverage change. Until 2015, the Registry covered Central Serbia only; from 2016, it covered Vojvodina as well. We checked this in three ways since no single check is sufficient on its own.

First, restricting the series to the years of consistent coverage, 1999–2015, the male trend disappears (APC = −0.30; *p* = 0.666). Second, modeling the 2016 step explicitly makes the step large and highly significant (+50.7%; *p* < 0.001) while the underlying trend is not (APC = −0.27; *p* = 0.657); the two approaches agree to within 0.03 percentage points. Third, a model with one trend and a step describes the male and combined series better than any continuous joinpoint model, despite using fewer parameters (Table 5).

We therefore do not read the significant male APC for 1999–2020, or the 2013–2020 acceleration, as evidence that oral cavity cancer is genuinely becoming more common among Serbian men.

A joinpoint model cannot distinguish a trend that changes direction from a series that jumps to a higher level, so it places the break wherever the line fits best. That is why it fell in 2013 for men and 2011 for both sexes, before the actual change. Even when allowed four joinpoints, the software never chose 2016: instead, it split the rise into a steep climb (APC = +14.30) and then a decline (−2.73), neither significant—a continuous line is the shape it must take to reproduce a step.

The confidence intervals around the break year show how imprecisely it is located. For both sexes, the best estimate is 2011, but the interval runs from 2005 to 2016—so the model does not exclude a break in 2016, the year coverage changed. In women, the interval spans seventeen years (2001–2018), meaning no break can be located at all. In men, the interval (2009–2015) stops short of 2016, but that is expected for the same reason.

The 2016 step in our data does, however, show something: oral cavity cancer is not evenly distributed within Serbia. Working back from the size of the step, incidence in Vojvodina must be roughly 2.6 times that of Central Serbia among men and 1.4 times among women. That is a wide gap but a believable one. Vojvodina borders Hungary, which has long recorded the highest oral and pharyngeal cancer rates in Europe [17,18], and rates across Central Europe decline towards the south-east [17,18]. Geographic differences of this kind within a single country are documented elsewhere: Radespiel-Tröger and colleagues found intraoral cancer incidence 34% higher in men and 25% higher in women in Upper Franconia, a socioeconomically disadvantaged region, than in Bavaria as a whole [38].

The difference implied by our data is considerably larger than that, and as far as we know has never been measured for the oral cavity in Serbia. That is a reason to measure it directly rather than infer it from a step in a national series. For a clinician, it nonetheless carries a simple message: a patient from Vojvodina belongs to a higher-risk population than one from Central Serbia.

Two consequences follow for the forecasts. First, the ARIMA models were built on the same mixed series and carry the same problem: the projected male level of 4.7 per 100,000 describes the combined territory after 2016, assuming coverage does not change again, and is not the continuation of a rising trend. Second, only five years of data follow the change, so that level rests on a short stretch of the series and is less firmly established than the model-based intervals suggest.

Once the coverage change is accounted for, the underlying trend is flat in both sexes, so in our data the sex gap neither widened nor narrowed. That is at odds with published projections by other authors [4,20]—which do not agree with each other either. Using Global Burden of Disease 2021 data, Mu and colleagues predict that by 2030 incidence will fall in men and rise in women, so that the gap narrows [4]. Chen and colleagues, using the same data, predict that rates will keep rising to 2040, with Eastern Europe among the worst affected regions [20]. When two forecasts built from the same data give opposite answers, neither can be applied to a single country with confidence—which is a practical reason to look at national registry data directly.

None of the explanations above could be tested directly because the Registry does not record whether a patient smoked, drank or carried HPV. Missing HPV data matters less for the oral cavity than it would for the oropharynx. HPV DNA is found in about a quarter of oral cavity cancers [13], but finding the virus is not the same as showing it caused the tumor. When expression of the oncogenes is required as well, Lingen and colleagues put the attributable fraction at about 6% [14], and pooled estimates place it near 3% [39]. Even so, we cannot tell whether any shift in HPV-related disease contributed to what we observed.

Analyzing C01–C06 as a single group is a further limitation, because trends at neighboring subsites can move in opposite directions. Registry data from 23 countries showed oral cavity cancer falling in most of them between 1983 and 2002 while oropharyngeal cancer rose, mainly in younger patients [40]. A national Scottish study for 2001–2020 confirms this within one population: oral cavity incidence was flat (−0.60% per year; *p* = 0.08), oropharyngeal cancer rose (+3.76%; *p* < 0.001), and laryngeal cancer fell (−2.56%; *p* < 0.001) [41]. Rising tongue cancer has also been reported in other national surveillance data [42]. None of these patterns would be visible in grouped figures such as ours.

The tongue is also the subsite where the sex difference behaves differently. Globally, the ratio is close to three to one (2.6 against 0.86 per 100,000) [43], wider than the ratio we found for the oral cavity as a whole—yet in New Zealand the tongue was the only one of four upper aerodigestive subsites in which incidence among older women exceeded that in men [44]. Our subsite data do not support the same pattern for Serbia: the tongue was, if anything, more male-dominant than the oral cavity overall (76% versus 68% male), while women outnumbered men only for tumors of other and unspecified mouth sites (Table 2)—a category too heterogeneous to interpret further without more detailed subsite coding.

The forecasts should be read as an indication of the general level to expect, not as a prediction for particular years. The model for women fitted poorly, with an average error of 32.9%, and two years dominate that series: 2000, with 140 cases, and 2003, with 156, two to three times the numbers recorded either side of them. Whether those years reflect something real or a reporting problem we cannot say, but they alone account for much of the year-to-year swing in the female data. The male forecast deserves caution in the opposite direction: it assumes the sharp climb of 2013–2020 does not continue, and if it does, our figures will prove too low.

This study has three strengths. It covers the largest part of the national population, it spans 22 years, and it uses two methods that answer different questions—joinpoint regression describes what has happened, ARIMA modeling indicates what may follow.

### Study Limitations

Several limitations remain. The most important is the change in Registry coverage in 2016: it is confounded with calendar time and cannot be separated from it using grouped data. Case numbers by region were not available to us, so we could not produce a single consistent series for the whole of present-day Serbia. Second, we studied populations rather than individuals, so nothing here settles the risk of any one patient. Third, grouping C01–C06 together hides differences between subsites, tumor types and stage at diagnosis. Fourth, projecting 20 years beyond the last observation is inherently uncertain, particularly for women. Fifth, in a country of this size the yearly case numbers are small enough, especially in women, that ordinary chance produces swings which can be mistaken for real change. Sixth, the incidence implied for Vojvodina is a counterfactual estimate that rests on an assumption about the unobserved post-2015 trajectory of Central Serbia; a sensitivity analysis relaxing this assumption (Table 4) shows the estimated gap narrows or widens somewhat depending on the trajectory assumed, but Vojvodina remains higher than Central Serbia under every scenario tested. Seventh, diagnostic and coding practices may have evolved over the 22-year study period—for example, improvements in histopathological verification or changes in ICD-10 coding conventions—and any such changes cannot be distinguished from genuine shifts in incidence using the data available to us. Finally, the Registry data do not include the Autonomous Province of Kosovo and Metohija in any year of the study period, so the rates reported here describe the remainder of the territory.

## 5. Conclusions

In the area the Registry covered consistently—Central Serbia up to 2015—oral cavity cancer incidence did not change appreciably. Recorded rates rose after 2015, but that rise coincides with the year in which the Registry began to cover Vojvodina as well, and three separate checks point to the change in coverage rather than to anything happening over time. Across the whole 22 years, the male rate was about twice the female rate. That gap is the one finding here that does not depend on how the coverage question is settled. The ARIMA projections show no material change through 2040, averaging about 2.6 per 100,000 overall and 4.7 in men; they describe the combined territory under unchanged coverage rather than predicting a rise in disease, and the projection for women is considerably less precise. If realized, a flat trajectory implies that the oral cancer caseload facing Central Serbian services is unlikely to grow materially over the next two decades under current conditions; given the widening prediction intervals, however, this should inform periodic re-evaluation of capacity rather than fixed long-term planning assumptions.

The size of the 2016 jump implies that incidence in Vojvodina is roughly 2.6 times that of Central Serbia among men. If that is correct, it is the most striking thing in these data, and it fits the known rise in tobacco- and alcohol-related cancers of the mouth and throat towards Hungary and Central Europe [17,18]. It also carries a direct clinical implication: where a patient comes from should count when deciding how carefully to examine the oral mucosa. A region-stratified study is needed to measure this properly.

For practice, three points follow from the established evidence rather than from our own data. Men who both smoke and drink remain the group at highest risk, and the two exposures multiply each other rather than simply adding up [11]. Risk does fall after stopping, but slowly: by about half after ten years, and only close to that of a lifelong non-smoker after twenty [5,7], which is why advice to quit is worth giving at any age. At the same time, about a quarter of head and neck cancers in men and more than four in ten in women are not attributable to tobacco or alcohol [11], so a suspicious lesion in someone who neither smokes nor drinks deserves exactly the same attention. Opportunistic visual and tactile examination of the oral mucosa during routine dental and primary care visits costs little and could plausibly be formalized as part of national cancer-control policy—for example, through structured screening protocols in dental facilities aimed at detecting both malignant and potentially malignant oral lesions [15]. Because our data are aggregated at the national level and lack information on stage at diagnosis or outcome, they cannot support recommendations for differential screening intensity by region; any such measures should be applied uniformly until region-stratified data become available.

Finally, this study is a reminder that registry data need consistent geographical coverage before national trends can be read from them. An administrative extension of surveillance can look exactly like an epidemiological trend: in our data, it produced a statistically significant annual rise of 8.39% among men, which disappears once the change is taken into account. The findings reported here stand firm: stable incidence in the consistently covered area, a persistent sex difference, and a marked internal geographic difference. What remains is to confirm the same picture for the whole Registry area, once case numbers by region become available.

## Figures and Tables

**Table 1:** Number of cases and age-standardized incidence rate (ASR, per 100,000) of malignant oral cavity tumors (ICD-10: C01–C06), by sex and year of diagnosis, Republic of Serbia, 1999–2020.

Year	Male (n)	Female (n)	Total (n)	ASR Male (95% CI)	ASR Female (95% CI)	ASR Total (95% CI)
1999	132	56	188	3.21 (2.67–3.76)	1.24 (0.91–1.56)	2.17 (1.86–2.49)
2000	169	140	309	4.24 (3.60–4.87)	3.82 (3.19–4.45)	3.98 (3.53–4.42)
2001	131	68	199	3.11 (2.58–3.65)	1.61 (1.22–1.99)	2.31 (1.99–2.63)
2002	132	48	180	3.22 (2.67–3.77)	0.98 (0.71–1.26)	2.05 (1.75–2.35)
2003	122	156	278	3.04 (2.50–3.57)	4.51 (3.80–5.21)	3.74 (3.30–4.18)
2004	127	57	184	2.91 (2.40–3.42)	1.41 (1.05–1.78)	2.11 (1.80–2.41)
2005	147	86	233	3.49 (2.93–4.06)	2.05 (1.62–2.48)	2.72 (2.37–3.07)
2006	150	67	217	3.58 (3.00–4.15)	1.33 (1.01–1.64)	2.39 (2.07–2.70)
2007	131	43	174	3.01 (2.49–3.52)	0.93 (0.65–1.21)	1.91 (1.62–2.19)
2008	154	51	205	3.58 (3.02–4.15)	1.11 (0.80–1.41)	2.27 (1.96–2.58)
2009	159	74	233	3.68 (3.11–4.26)	1.63 (1.26–2.00)	2.59 (2.26–2.93)
2010	135	55	190	3.15 (2.62–3.68)	0.89 (0.66–1.13)	1.97 (1.69–2.25)
2011	108	48	156	2.49 (2.02–2.96)	0.95 (0.68–1.22)	1.68 (1.42–1.95)
2012	147	68	215	3.25 (2.73–3.78)	1.69 (1.29–2.09)	2.42 (2.09–2.74)
2013	109	93	202	2.60 (2.11–3.09)	1.89 (1.51–2.28)	2.23 (1.92–2.54)
2014	164	64	228	3.68 (3.12–4.25)	1.18 (0.89–1.47)	2.37 (2.06–2.67)
2015	164	56	220	3.76 (3.19–4.34)	1.10 (0.81–1.38)	2.36 (2.05–2.67)
2016	269	120	389	4.40 (3.87–4.92)	1.77 (1.45–2.09)	3.00 (2.71–3.30)
2017	309	125	434	5.00 (4.45–5.56)	1.69 (1.39–1.98)	3.24 (2.93–3.54)
2018	304	103	407	5.00 (4.44–5.56)	1.35 (1.09–1.61)	3.09 (2.79–3.38)
2019	314	129	443	4.97 (4.42–5.51)	1.56 (1.29–1.82)	3.17 (2.87–3.46)
2020	295	116	411	4.55 (4.03–5.07)	1.35 (1.11–1.60)	2.85 (2.58–3.13)

CI—confidence interval. Data cover Central Serbia for 1999–2015, and Central Serbia together with Vojvodina from 2016 onwards; the Autonomous Province of Kosovo and Metohija is not covered in any year. Rates are therefore not directly comparable across the 2016 boundary.

**Table 2:** Distribution of malignant oral cavity tumors (ICD-10: C01–C06) by anatomical subsite and sex, Republic of Serbia, 1999–2020.

Total, n (%)	Female, n (%)	Male, n (%)	Subsite (ICD-10)
2469 (43.4)	591 (23.9)	1878 (76.1)	Tongue (C01–C02)
310 (5.4)	111 (35.8)	199 (64.2)	Gum (C03)
1156 (20.3)	279 (24.1)	877 (75.9)	Floor of mouth (C04)
549 (9.6)	185 (33.7)	364 (66.3)	Palate (C05)
1211 (21.3)	657 (54.3)	554 (45.7)	Other/unspecified mouth (C06)
5695 (100)	1823 (32.0)	3872 (68.0)	Total

Male/Female percentages are row percentages (% within subsite); total column is % of grand total. χ^2^ test of association between subsite and sex: χ^2^ = 74.2, *p* < 0.001.

**Table 3:** Sensitivity analyses of the estimated annual percentage change in malignant oral cavity tumor incidence, accounting for the extension of Registry coverage in 2016.

Sex	Full Series 1999–2020 APC (95% CI)	Consistent Coverage 1999–2015 APC (95% CI)	Level-Shift Model 1999–2020 APC (95% CI)	Level Shift at 2016 (95% CI)
Male	1.71 * (0.47, 2.96)	−0.30 (−1.63, 1.06)	−0.27 (−1.48, 0.94)	+50.7% * (25.5, 81.1)
Female	−1.43 (−4.21, 1.43)	−3.31 (−7.41, 0.96)	−3.38 (−7.00, 0.38)	+51.5% (−15.0, 170.2)
Both	0.56 (−1.00, 2.14)	−1.43 (−3.43, 0.60)	−1.43 (−3.20, 0.37)	+51.6% * (15.3, 99.3)

APC—annual percent change; CI—confidence interval. * statistically significant (*p* < 0.05). The level-shift model estimates a common underlying annual trend together with a multiplicative change in level from 2016 onwards, when Registry coverage was extended from Central Serbia to include Vojvodina (see Table 1).

**Table 4:** Sensitivity of the counterfactual Vojvodina incidence estimate to the assumed post-2015 trajectory of Central Serbia, 2016–2020.

Ratio	Implied Vojvodina Rate	Central Serbia Rate, 2016–2020	Central Serbia Trajectory Assumption
2.6×	8.7	3.4	Men: frozen at 2013–2015 level (primary estimate)
2.1×	7.8	3.7	Men: consistent-coverage trend, point estimate (−0.30%/yr)
2.3×	8.2	3.6	Men: consistent-coverage trend, lower 95% CI (−1.63%/yr)
1.9×	7.4	3.9	Men: consistent-coverage trend, upper 95% CI (+1.06%/yr)
2.2×	5.2	2.3	Both sexes: frozen at 2013–2015 level (primary estimate)
2.4×	5.4	2.3	Both sexes: consistent-coverage trend, point estimate (−1.43%/yr)
2.7×	5.7	2.1	Both sexes: consistent-coverage trend, lower 95% CI (−3.43%/yr)
2.1×	5.0	2.4	Both sexes: consistent-coverage trend, upper 95% CI (+0.60%/yr)

Rates are per 100,000 (world age-standardized). The primary estimate assumes Central Serbia’s rate remained at its 2013–2015 mean after 2015; sensitivity rows instead project the Central Serbia rate forward from 2015 using the point estimate or 95% confidence bounds of the consistent-coverage (1999–2015) joinpoint annual percentage change. Under every trajectory tested, the counterfactual Vojvodina rate exceeds that of Central Serbia.

**Table 5:** Comparison of three descriptions of the age-standardized incidence series, by Bayesian Information Criterion (BIC). Lower values indicate a better description.

Series	Single Log-Linear Trend	Best Continuous One-Joinpoint Model	Trend Plus Level Shift at 2016
Both sexes	−2.8191	−2.9453 (break 2011)	−3.0622
Male	−3.3018	−3.7131 (break 2013)	−3.8591
Female	−1.6062	−1.4442 (break 2010)	−1.5650

BIC—Bayesian Information Criterion. The best description of each series is shown in bold. The break position in the one-joinpoint model was selected by exhaustive search over all candidate years, and coincided with the position returned by the Joinpoint software in every series. The level-shift model uses one parameter fewer than the one-joinpoint model. BIC values in this table are computed on log-transformed rates and are not comparable with those reported for the ARIMA models (Section 3.4).

**Table 6:** Joinpoint regression analysis of malignant oral cavity tumor (ICD-10: C01–C06) incidence trends, by sex, Republic of Serbia, 1999–2020.

Sex	Period	APC	95% CI	*p*-Value
Male + Female	1999–2020	0.56	−1.00, 2.14	0.465
Male + Female	1999–2011	−2.57	−5.77, 0.74	0.118
Male + Female	2011–2020	5.48 *	0.18, 11.05	0.043
Male	1999–2020	1.71 *	0.47, 2.96	0.009
Male	1999–2013	−0.64	−2.26, 1.00	0.420
Male	2013–2020	8.39 *	3.44, 13.57	0.002
Female	1999–2020	−1.43	−4.21, 1.43	0.306
Female	1999–2010	−5.13	−12.64, 3.02	0.195
Female	2010–2020	2.99	−6.36, 13.28	0.522

APC—annual percent change; CI—confidence interval. * statistically significant (*p* < 0.05). A maximum of four joinpoints was permitted. Estimated joinpoint locations (95% CI): both sexes 2011 (2005, 2016); men 2013 (2009, 2015); women 2010 (2001, 2018). Coverage was extended from Central Serbia to include Vojvodina in 2016 (see Table 1).

**Table 7:** Validation parameters of the fitted ARIMA models used to forecast the age-standardized incidence of malignant oral cavity tumors (ICD-10: C01–C06), by sex.

Sex	ARIMA (*p*,*d*,*q*)	AICc	BIC	Ljung–Box *p*	Shapiro–Wilk *p*	RMSE	MAPE (%)	MAE
Both sexes	(0,0,0)	43.0	44.5	0.336	0.145	0.578	18.6	0.474
Male	(1,1,0)	38.8	40.3	0.796	0.512	0.531	13.2	0.462
Female	(0,0,0)	60.7	62.3	0.393	<0.001	0.866	32.9	0.541

AICc—corrected Akaike Information Criterion; BIC—Bayesian Information Criterion; RMSE—root mean square error; MAPE—mean absolute percentage error; MAE—mean absolute error. The 95% prediction interval for each forecast is reported in the text above and shown as shaded bands in Figure 4, Figure 5 and Figure 6.

## Data Availability

Restrictions apply to the availability of these data. The data were obtained from the Cancer Registry of the Republic of Serbia, Institute of Public Health of Serbia “Dr Milan Jovanović Batut”, and are available from the Registry with its permission. The aggregated annual case counts and age-standardized rates supporting the reported results are contained within the article (Table 1).

## Supplementary Materials

The following supporting information can be downloaded at: https://www.mdpi.com/article/10.3390/medicina62091757/s1, Table S1: Annual case counts, population denominators, and age-standardized incidence rates (ASR) for malignant oral cavity tumors (ICD-10: C01–C06), Republic of Serbia, 1999–2020, by sex; Code S1: Python script (statsmodels) reproducing the ARIMA models reported in Table 7, including the sensitivity analysis excluding the anomalous 2000 and 2003 values for women.
