# Supplementary material for: Incidence of Malignant Oral Cavity Tumors in the Republic of Serbia: Trend Analysis (1999–2020) and Projections to 2040
Source: Medicina (Kaunas). 2026 Sep 11;62(9):1757. doi: 10.3390/medicina62091757 (PMC13609485; doi:10.3390/medicina62091757)
Supplement: Supplementary file 1 [file medicina-62-01757-s001.zip › README_Supplementary_Materials.pdf]

Supplementary Materials

Incidence of Malignant Oral Cavity Tumors in the Republic of Serbia: Trend Analysis (1999–2020) and Projections to 2040

Prepared in response to Reviewer 2 (minor comment): *“Provide annual case counts, population denominators, and statistical code as supplementary materials.”*

Contents

Supplementary Table S1 (`Supplementary_Table_S1_annual_data.csv`) Annual case counts, population denominators, and age-standardized incidence rates (ASR, world standard population) for malignant oral cavity tumors (ICD-10: C01–C06, excluding lip), Republic of Serbia, 1999–2020, by sex.

Column	Description
`year`	Calendar year
`coverage_area`	Registry coverage area for that year (Central Serbia, 1999–2015; Central Serbia + Vojvodina, 2016–2020)
`cases_male`, `cases_female`, `cases_total`	Registered case counts
`population_male`, `population_female`, `population_total`	Resident population denominators used to calculate ASR
`asr_male_per_100000`, `asr_female_per_100000`, `asr_both_per_100000`	Age-standardized incidence rate (world standard population), per 100,000, as reported in Table 1 of the manuscript

Case counts and ASR values are reproduced from manuscript Table 1. Population denominators were extracted from the authors’ original age-standardization worksheet and independently validated: recomputing the world-standardized rate from the age-group-specific population and case data reproduces the published 1999 ASR values (male 3.21, female 1.24, both sexes 2.17) to within rounding.

Supplementary Code S1 (`Supplementary_Code_S1_ARIMA_models.py`) Python script (statsmodels) that fits the three primary ARIMA models reported in Table 7 (both sexes, men, women) and the sensitivity model for women excluding the anomalous 2000 and 2003 values (see Discussion), reporting model orders, AIC/BIC, validation statistics, and 95% prediction intervals through 2040. Requires `pandas`, `numpy`, `statsmodels`, `scipy`.

Note on population denominators

Annual population denominators for Vojvodina alone are not separately available; the Registry's published rates (and this study's) are calculated against Central Serbia alone (1999–2015) and Central Serbia plus Vojvodina combined (2016–2020), as described in Methods, Section 2.1. The counterfactual incidence estimated for Vojvodina (Results, Section 3.2) is derived algebraically from the combined-area rate and the Vojvodina population weight (26%), not from a directly observed Vojvodina-specific denominator.
